# Structural dynamics in the La-module of La-related proteins

**DOI:** 10.1080/15476286.2020.1733799

**Published:** 2020-03-18

**Authors:** Javier Lizarrondo, Anne-Catherine Dock-Bregeon, Luigi Martino, Maria R Conte

**Affiliations:** a Randall Centre for Cell & Molecular Biophysics, King’s College London, Guy’s Campus, London, UK; b Laboratoire De Biologie Intégrative Des Modèles Marins, Station Biologique De Roscoff, CNRS-Sorbonne Université, Roscoff, France; c The Francis Crick Institute, Molecular Structure of Cell Signalling Laboratory, London, UK

**Keywords:** La-related proteins, La-module, RNA recognition, SAXS, structural dynamics

## Abstract

The La-related proteins (LaRPs) are a superfamily of eukaryotic RNA-binding proteins with important and varied roles. To understand LaRP functions it is essential to unravel the divergent features responsible for their RNA target selectivity, which underlie their distinct identities and cellular roles. LaRPs are built on a common structural module called the ‘La-module’ that acts as a main locus for RNA recognition. The La-module is comprised of two tethered domains whose relative structural and dynamic interplay has been proposed to regulate RNA-target selection, albeit the mechanistic underpinning of this recognition remains to be elucidated. A main unsolved conundrum is how conserved La-modules across LaRPs are able to bind to extremely diverse RNA ligands.

In this work, we employed Small Angle X-ray Scattering (SAXS) to investigate several human LaRP La-modules in the absence and, where applicable, in the presence of their RNA target, with the aim to explore the structural dynamics of their RNA recognition and provide information on the architectural landscape accessible to these proteins. Integration of these SAXS experiments with prior X-ray crystallography and NMR data suggests that RNA binding is generally accompanied by a compaction and loss of flexibility of the La-module. Nonetheless, the La-modules appear to experience a considerably different degree of inherent flexibility in their apo state. Furthermore, although they all exist in discrete subsets of accessible populations in equilibrium, these vary from LaRP to LaRP and can be either extended or compact. We propose that these divergent features may be critical for RNA substrate discrimination.

## Introduction

The La-related proteins (LaRPs) constitute a wide and diverse superfamily of RNA-binding Proteins (RBPs) with assorted and important functions in the regulation of gene expression [1,2]. The archetype of this family, the La protein, was first identified in the mid-seventies as an autoantigen in autoimmune disorders including lupus erythematosus and Sjogren’s syndrome [3], and intensively studied since [2,3]. On the contrary, the majority of the other LaRPs have only been discovered and classified in the last decade [1]. The LaRPs have been evolutionary grouped into 5 families, dubbed LaRP1, LaRP3 (La), LaRP4, LaRP6 and LaRP7 and share a highly conserved winged-helix domain, the La-Motif (LaM), appended by an RNA Recognition Motif (RRM1) [1,2]. Beyond this, the organization of each LaRP family diverges, with additional family-specific domains and sequences [2]. The tandem arrangement of the LaM and the RRM1 constitutes the La-module, a novel RNA-binding platform initially discovered in La, and conserved across most (if not all) LaRPs [2,4]. Evolutionary analysis of the La-module revealed that both LaM and RRM1 domains co-evolved, plausibly for fine-tuning towards specific RNA substrate selection [1]. In human (Hs) La, LaRP7 and LaRP6, the LaM and the RRM1 have been demonstrated to act cooperatively to recognize their RNA targets, and this synergic mode of binding is deemed to be recapitulated in most LaRPs [2,4–7].

Within the La-module, the LaM is highly conserved while the RRM1 domains exhibit family- and member-specific traits [2,8]. The linkers connecting the LaM and RRM1 have also been signalled as a highly divergent portion of the La-module across LaRPs, exhibiting different lengths and sequences [2,7,8], albeit the delineation of their boundaries is far from straightforward, especially when structural data are absent and uncertainties on domain assignment exist [9] (see below). The LaM contains six residues (Q20, Y23, Y24, D33, F35 and F55 – HsLa numbering will be used throughout unless specifically stated) that decorate its hydrophobic pocket and are essential for specific RNA binding *via* stacking interactions, H-bonds and electrostatic contacts. In La and LaRP7, D33 determines a 3ʹ-OH-dependent recognition mode. These six residues have been found conserved in all LaRPs except for the LaRP4 family and some plant LaRP6 members, where the Y24 and F55 positions diverge [2,10].

Despite the evolutionary conserved features, the LaRP La-modules recognize rather different RNA targets, varying in length, structure and nucleotide sequence [2]. For instance, whereas La binds to 3–4 nt single-stranded oligoU stretch with a 3ʹ termini mode of recognition, LaRP6 interacts in a 3ʹOH independent manner with a highly conserved stem-loop (SL) element in the 5′ UTR of α1(I), α2(I) and α1(III) collagen mRNAs, denoted hereafter as 48ntSL RNA. Even within the same family, members can display distinct RNA-binding properties, as exemplified by the three *Arabidopsis thaliana* LaRP6 proteins (AtLaRP6A, AtLaRP6B and AtLaRP6C) that differ from one another and from the eutherian counterparts ([7,10] and Conte, unpublished). The La and LaRP7 families share the highest similarity: their La-modules both recognize 3ʹUUU_OH_ sequences, but whereas La binds to the 3ʹ oligoU trailer of all RNA polymerase III transcripts, ensuring their correct folding and maturation, LaRP7 binds to the 3ʹUUU_OH_ 7SK RNA, a nuclear non-coding RNA (snRNA) involved in regulation of the positive transcription elongation factor b (pTEFb) [2,6,11]. LaRP7 selectivity for 7SK RNA is provided by a second RRM, located in the C-terminal region, which binds to a conserved hairpin in this RNA target ([6] and www.pnas.org/cgi/doi/10.1073/pnas.1806276115).

In the LaRP4 family, an early gene duplication gave rise to the LaRP4A and LaRP4B lineages and a neofunctionalisation event accompanied by the acquisition of a PAM2 motif resulted in a less conserved LaM [10]. Both LaRP4A and LaRP4B enhance protein translation and stabilize mRNAs, albeit binding to different RNA sequences. To date, the RNA target of HsLaRP4A has been identified as the 3ʹ polyA tail of mRNAs [12], although potential new targets have started to emerge [13]. HsLaRP4B interacts with AU-rich regions in the 3ʹUTR of a subset of mRNAs [14].

Reconciling the highly conserved features of the LaRP La-modules with their different RNA-binding properties and specificities remains a conundrum. With limited structural and dynamics information, many questions remain open on the molecular mechanism of RNA recognition by the La-modules and their determinants of RNA target discrimination [2,8]. The functional significance of the conserved six residues is one of the many unresolved mysteries: how can the same residues be involved in specific RNA contacts with different RNA substrates? Human La is the best characterized system to date: a crystal structure of the La-module in complex with 3ʹUUU_OH_ reveals a V-shaped arrangement with the LaM and the RRM1 accommodating the RNA in a binding site formed by the hydrophobic pocket of the LaM and the tandem domain cleft interface [5,15,16]. The LaM and RRM1 are structurally and motionally independent and do not adopt a fixed orientation with respect to one another in the apo state, as evinced by NMR investigations [4,5,17]. The linker connecting the two domains displays flexibility in the RNA-free state but rigidifies into a helical structure in the RNA-bound form, thereby playing a topological role in orienting the LaM and RRM1 in the compact configuration competent for RNA binding [5,15,17]. Furthermore, hydrogen-bonding contact between the side chains of Y23 in the LaM and N139 in the RRM1 appears critical for tandem domain alignment in the complex with RNA [5,16]. The crystal structure of HsLaRP7 La-module bound to 3ʹUUU_OH_ reveals protein-RNA contacts and modular domain characteristics similar to HsLa [6], as extensively reviewed in [8]. Although the interdomain linker of HsLARP7 La-module is shorter than in HsLa, in the complex with UUU_OH_ it also adopts a helical fold [6], likely contributing to the correct positioning of the LaM and RRM1 to present the V-shaped RNA-binding platform. Interestingly, interdomain hydrogen-bonding contact is also observed in HsLaRP7-U4 complex, involving K53 and E172 [6]. Regrettably, the lack of information of HsLaRP7 La-module in the apo form prevents the analysis of conformational changes experienced by linker and/or by individual domains upon RNA interaction.

Beyond HsLa and HsLaRP7, no structural information exists for other La-modules in complex with RNA. The structure of the La-module of HsLaRP4A in the apo state was recently solved by NMR spectroscopy, reporting relative conformational flexibility of the LaM and the RRM1 [18]. The short linker connecting the two domains coupled with the lack of the so-called ‘wing2ʹ of the LaM – the last loop extending from strand β3, characteristic of winged-helix domains [2,4] – imposes a more elongated spatial arrangement that appears distinct from that of HsLa and HsLaRP7 [18]. Unexpectedly, the La-module of HsLaRP4 only plays a peripheral role in RNA recognition, at least for the single-stranded polyA target, hinting at a possible relationship between the RNA-binding capability of the La-module and its tandem architecture [18]. Although a structure for HsLaRP6 La-module is not yet available, investigations of its isolated domains uncovered a short LaM-RRM1 interconnecting linker and a somewhat different exit path of the LaM resulting from variations in its wing2 loop [7]. Replacing the short linker of HsLaRP6 with the longer one from HsLa resulted in a 10-fold decreased RNA-binding affinity [7], inferring a clear role of the LaM-RRM1 linker in RNA recognition. Whilst awaiting for further molecular details, it can be envisioned that in HsLaRP6 the short linker may restrict the maximum distance between the LaM and RRM1 and/or regulate interdomain geometry and dynamics in both the apo and bound state.

Although current data argue that a correct combination of LaM, linker and RRM1 is needed to achieve the desired RNA-binding affinity and specificity, the mechanistic underpinning of this recognition remains to be elucidated. This would require a structural knowledge of both the isolated species and the protein-RNA complexes, coupled with conformational dynamics data of LaM, RRM1 and linker in the free and bound states, to ascertain their exact roles in the mechanism of complex formation [19].

In the present study we investigate La-modules of five human LaRPs in the apo and, where applicable, RNA-bound states using Small Angle X-ray Scattering (SAXS) and combined these data with existing structural and dynamics information to shed light on the pathway of protein-RNA complex formation. SAXS is a robust and versatile methodology for characterizing flexibility and shape of biomolecules, thereby providing insights into the conformational properties of multi-domain complex systems from a single experiment in native conditions [20,21]. These analyses provided information on flexibility and conformational ensemble distributions of La-modules in the apo and holo states. In particular, our data show that RNA recognition is generally accompanied by a rigidification of the La-module. They also revealed that the unbound La-modules sample the conformational space and exist in discrete subsets of accessible populations, suggesting that conformational selection may play a role in RNA substrate recognition for some of the La-modules of LaRPs.

## Materials and methods

### Protein purification

The La-modules of HsLa, HsLaRP7, HsLaRP6 and HsLaRP4A were all expressed in *E.coli* BL21 *DE3 orRosetta II* strain as described previously [5–7,18]. HsLaRP4B La-module was expressed in *Rosetta II*. The exact constructs used were as follows: HsLa (4–194); HsLaRP7 (1–208); HsLaRP6 (three variants spanning residues 70–300, 74–300 and 85–300, respectively); HsLaRP4A (111–287) and HsLaRP4B (151–328). The domain boundaries for each La-module were determined by structural investigations ([5–7,18] and unpublished). The isolated LaM and RRM1 domains of HsLaRP6 and HsLaRP4A were also prepared as previously described [22,23], and their domain boundaries were derived from prior structural analyses [22,23].

All the protein samples were purified following a three-step purification protocol consisting of an IMAC Ni^2+^ affinity step (His-Trap FF, GE Healthcare), removal of the N-terminal His-tag using either TEV (Tobacco Etch virus) protease or thrombin digestion overnight, a gravity Ni-NTA column for the removal of the non-cleaved tagged protein and proteases, followed by a Hi-Trap Heparin or DEAE chromatography (GE Healthcare), as described previously [5,7,22,23]. Finally, proteins were dialysed overnight in a final buffer containing 20 mM Tris pH 7.25, 100 mM KCl and 1 mM Dithiothreitol (DTT) for HsLa, HsLaRP4A, HsLaRP4B and HsLaRP6, or 20 mM HEPES pH 7.2, 200 mM NaCl, 5 mM MgCl_2_ and 0.5 mM TCEP (tris(2-carboxyethyl)phosphine) for HsLaRP7. Purified protein samples were concentrated and flash frozen in liquid nitrogen or used without further manipulation.

### RNA oligos

The 48ntSL of the 5ʹ UTR of α1(I) collagen mRNA [7] and 4 nt oligoU (U4) RNAs were purchased from IBA (IBA GmbH, Germany). The lyophilized RNA was resuspended in diethyl pyrocarbonate (DEPC)-treated water. The RNA concentration was evaluated by UV measurement at room temperature using the appropriate molar extinction coefficients at 260 nm [5,7].

### SEC-SAXS (size exclusion chromatography-small angle x-ray scattering) data acquisition

SEC-SAXS data were collected at the SOLEIL Light Source on beamline SWING. Samples at a concentration of around 150–200 μM were loaded onto a size exclusion column (Agilent BioSEC3) with a pore size of 300 Å, previously equilibrated in 20 mM Tris pH 7.5, 100 mM KCl, 1 mM TCEP or 20 mM HEPES pH 7.2, 200 mM NaCl, 5 mM MgCl_2_ and 0.5 mM TCEP for HsLaRP7. The main advantage of SEC-SAXS is that it allows the separation of monodisperse samples from aggregates and from any excess RNA ligand used to prepare the LaRP-RNA complexes. SEC-SAXS data were acquired for the following species: La-modules of HsLa, HsLaRP7, HsLaRP6, HsLaRP4A, HsLaRP4B; isolated LaM and RRM1 domains of HsLaRP6 and HsLaRP4A; complexes of HsLa, HsLaRP7 and HsLaRP6 La-modules with cognate RNAs (U4 and 48ntSL, respectively). For the complexes, the RNAs (U4 or 48ntSL) were incubated with the protein at an RNA:protein molar ratio of 1.2–1.5. For HsLaRP6 La-module, three fragments were tested, spanning residues 70–300, 74–300 and 85–300, respectively (Supplementary Fig. S1). The region between residues 70 and 84 was found to be mostly unstructured in our previous investigations [7], hence varying N-terminal boundaries was intended to improve protein behaviour and reduce the tendency to aggregate in solution. Although these mutants showed similar behaviour in solution (Supplementary Fig. S1) and retained comparable RNA-binding capability towards the 48ntSL collagen RNA [7], the fragment 85–300 was selected for the subsequent study, to limit possible complications in the SAXS interpretation and analysis arising from the flexible N-terminal region.

### SEC-SAXS data reduction and analysis

The primary reduction of the SAXS data was performed using the Foxtrot software from the SWING beamline at SOLEIL synchrotron (https://www.synchrotron-soleil.fr/en/beamlines/swing). Briefly, buffer curves were averaged and used to correct for the solvent effect on the SAXS data of the elution profiles. Then, an initial Guinier approximation was employed to obtain the radius of gyration (R_g_) of each frame along the elution profiles. Curves showing a constant R_g_ in the elution profile were averaged (they generally correspond to the frames in the middle of the elution peak). This allowed the extraction of a SAXS data curve for each sample, corresponding to the average of solvent-corrected data curves showing a constant R_g_ (Supplementary Fig. S2), using the following frame numbers: 275–280 for HsLa, 270–290 for La-U4 complex, 327–353 for HsLaRP7, 354–379 for HsLaRP7-U4 complex, 235–255 for HsLaRP6, 215–230 HsLaRP6-48ntSL complex, 230 − 250 for HsLaRP4A and 360–380 for HsLaRP4B.

Data processing was carried out with the ATSAS package version 2.8.4 [24]. PRIMUS [25] was used to obtain R_g_, the maximum particle dimension (D_max_), the excluded particle volume (V_POROD_) while GNOM (run under PRIMUS) was used to evaluate the pair distribution function (P(r)). As the GNOM-derived Total Quality Estimate values for the P(r) are all close to the unit (Supplementary Table S1), the chosen functions are all classified as good/excellent GNOM solutions. A similar analysis was also performed with SCATTER [26] to check whether the parameters obtained were reproducible using complementary protocols. Low resolution three-dimensional *ab-initio* models were generated using the program DAMMIF [27] and averaging the results of 25 independent DAMMIF runs was done using DAMAVER [28]. CRYSOL [29] was employed to compare prior high-resolution structures with the experimental scattering profiles. The high-resolution structures were fit into the generated *ab-initio* models with SUPCOMB [30]. Ensemble optimization method (EOM) [31] was used to assess the relative domain orientation of the LaM and RRM1 in the context of the La-modules. In the EOM pipeline, a stochastic genetic algorithm was used to generate a total of 10,000 models covering a diverse set of conformations of the La-modules that would reflect the spatial orientations of the LaM and the RRM1 domains obtained by leaving the linker residues to move freely. Specifically, the linker residues left unrestrained based on the prior knowledge of the structures and NMR dynamics, were: 99–107 for HsLa, 117–120 for HsLaRP7, 177–180 for HsLaRP6 and 197–199 for HsLaRP4A. Where appropriate, the N and C-terminal residues were also modelled as dummy residues (residues 1–28 and 189–208 for HsLaRP7, 296–300 for HsLaRP6 and 275–287 for HsLaRP4A). The stochastically calculated models populate compact and extended conformations, following a normal distribution profile. The initial conformation pool was filtered against experimental SAXS data to find the best representative ensemble of structures with the lowest discrepancy fit to the experimental data. The structures and *ab-initio* models were shown using PyMol (https://pymol.org/2/).

## Results

### SEC-SAXS analysis of the apo HsLa, HsLaRP7, HsLaRP6, HsLaRP4a and HsLaRP4b La-modules

**Figure 1.**
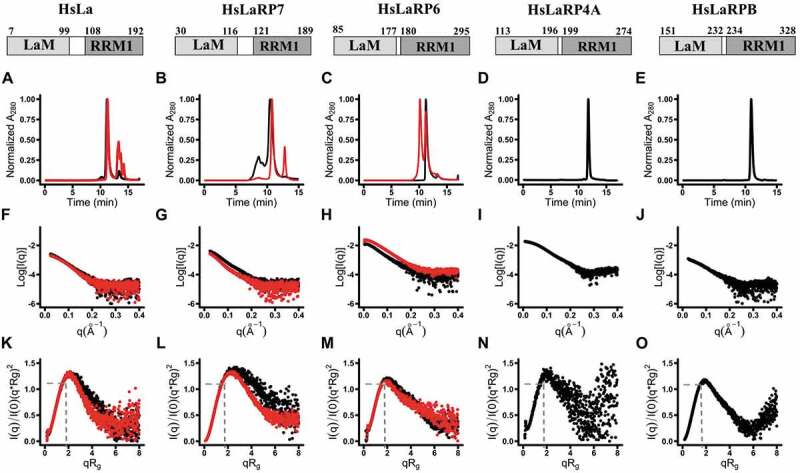
Small Angle X-ray Scattering analysis of HsLa, HsLaRP7, HsLaRP6 and HsLaRP4A and HsLaRP4B La-modules. The domain boundaries of the La-motif (LaM) and RNA Recognition Motif 1 (RRM1), delineating the exact beginning and end of the structured domains, are indicated on top for each protein. In the case HsLaRP4B, for which structural analysis is underway (Conte et al., unpublished), this is an initial estimate from sequence alignment with LaRP4A proteins. (A-E) SEC elution profiles for the five La-modules in the apo state as labelled (black traces). The SEC elution profiles for HsLa, HsLaRP7 and HsLaRP6 in complex with U4 for HsLa and HsLaRP7 and with 48ntSL RNA for HsLaRP6 are shown as red traces in A, B and C. (F-J) Scattering curves obtained after buffer normalization and averaging (black traces for the apo La-modules, red traces for the complexes with RNA). (K-O) Normalized Kratky representations (in black for the apo La-modules and in red for the complexes with RNAs) calculated from data in the range q = 0.02–0.3. The typical values expected for globular proteins [I(q)/I(0)]⋅(q⋅R_g_)^2^ = 1.104, q⋅R_g_ = 1.73] are indicated by grey dashed cross lines

To gain new insights into the mechanism of RNA recognition of the La-module from different LaRPs, we have embarked on a comparative study using in-line Size Exclusion Chromatography coupled to Small Angle X-ray Scattering (SEC-SAXS) on HsLa, HsLaRP7, HsLaRP6, HsLaRP4A and HsLaRP4B. For these proteins, previous studies have delineated the exact domain boundaries for the La-modules, LaMs and RRM1s [4–7,18,22,23] (and Conte unpublished) (Fig. 1). First, we examined the data for the apo La-modules (Fig. 1, black curves): in the absence of RNA, most La-modules migrate as single monodisperse species on the size exclusion column (black traces in Fig. 1A–E), but HsLaRP7 showed some aggregation at the concentration used in this experiment (around 200 µM). Averaged SAXS curves corrected for the solvent effect were obtained as reported in the methods (Fig. 1F–J, Supplementary Fig. S2).

**Figure 2.**
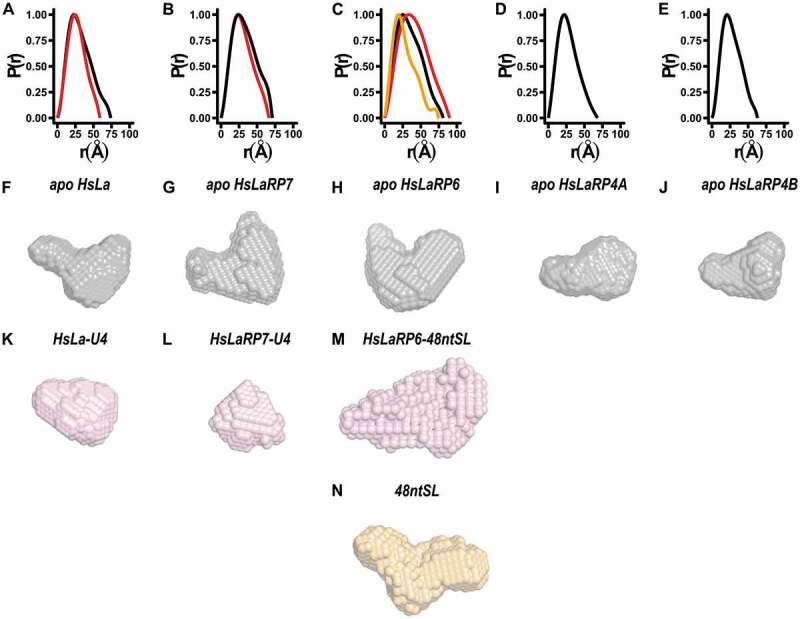
Distance distribution functions and *ab-initio* models. (A-E) Distance distribution functions for the La-modules of La, HsLaRP7, HsLaRP6, HsLaRP4A and HsLaRP4B in the apo form (black traces) and for the La-modules of HsLa, HsLaRP7 and HsLaRP6 in complex with their target RNAs in red traces. The distance distribution function for free 48ntSL RNA is shown in orange in C. (F-N) Low-resolution *ab-initio* models were generated from the distance distribution functions for the La-modules (F-J, grey), for HsLa and HsLaRP7 and HsLaRP6 in complex with RNA (K-M, red) and for the 48ntSL RNA (N, orange). A similar orientation for each model was chosen (approximately as in Fig. 4A, left) based on superposition on the HsLa structure following SUPCOMB fitting of the *ab-initio* models to the respective atomic structures, when available, or to the HsLa structure for HsLaRP6 and HsLaRP4B

The analysis of the normalized Kratky plots (Fig. 1K–O) suggests that the La-modules populate non-globular conformations in solution, as evinced by the asymmetric shape of the plots and by the fact that in all the cases the maxima are shifted from the typical values expected for globular proteins [I(q)/I(0)]⋅(q⋅R_g_)^2^ = 1.104, q⋅R_g_ = 1.73] [32] (Fig. 1K–O). Of the La-modules analysed, the most globular appear to be HsLaRP6 and HsLaRP4B. Furthermore, a degree of intrinsic flexibility is manifest in all the curves, in that the regions at high value of q⋅R_g_ do not return back to zero, deviating from a normal distribution profile [33,34]. This up-turn of the Krakty plot is however particularly pronounced for HsLaRP4A and HsLaRP7, whilst HsLaRP4B appears the least flexible La-module, with its normalized Kratky representation more closely resembling a normal distribution. The Porod-Debye representation supports these conclusions, with the Porod plateau reached sooner for HsLa, HsLaRP6, HsLaRP4B and HsLaRP7 compared with HsLaRP4A, denoting greater flexibility in the latter [34] (Supplementary Fig. S3). Radii of gyration (R_g_) were obtained through the Guinier analysis of the low-q value region of the curves, while values of maximum distances (D_max_) and Volume of Porod (V_POROD_) were evaluated by the distance distribution function (P(r)) (Fig. 2, Table 1). These parameters enabled the evaluation of the SAXS-derived molecular weights for each La-module, and these agree well with values expected from primary sequences (Table 1, Supplementary Table S1).

**Table 1.** SAXS-derived parameters and experimental details

Instrument		SEC-SAXS at SWING beamline SOLEIL						
q range (Å ^−1^)		0.0022–0.62						
Temperature (°C)		25°C						
Sample	R_g_(Å) Reciprocal Space	I_(0)_ (cm^−1^/absorbance) Reciprocal Space	R_g_(Å) Real Space	I_(0)_ (cm^−1^/absorbance) Real Space	D_max_ (Å)	Porod Volume estimate (Å^3^)	MW from Porod Volume/ 1.6 (kDa)	MW from sequence (kDa)
HsLa La-module	26.90 ± 0.40	00029 ± 1.1 10^−5^	26.97 ± 0.09	0.002876 ± 8.285 10^−6^	84	46400	29.0	22.3
HsLa La-module-U4 complex	23.82 ± 0.25	0.0022 ± 1.5 10^−5^	22.76 ± 0.08	0.002013 ± 6.678 ± 10 ^−6^	69	40300	25.0	22.3
HsLaRP7 La-module	32.08 ± 1.02	0.013 ± 2.6 10^−5^	32.20 ± 0.10	0.01289 ± 2.965 10^−5^	101	58200	36.4	24.0
HsLaRP7 La-module-U4 complex	26.79 ± 0.30	0.024 ±3.5 10^−5^	27.40 ± 0.05	0.02369 ± 3.196 10^−5^	92	48000	30.0	24.0
HsLaRP6 85–300 La-module	27.18 ± 0.18	0.0045 ± 3 10^−5^	27.20 ± 0.07	0.004412 ± 1.539 10^−5^	81	51900	32.4	23.9
HsLaRP6 85–300 La-module-48ntSL complex	31.48 ± 0.13	0.0032 ± 1.8 10^−6^	31.60 ± 0.07	0.003138 ± 1.220 10^−5^	90	94000	58.8	39.8
48ntSL RNA	22.29 ± 1.77	0.0091 ± 9.9 10^−6^	22.47 ± 0.03	0.009076 ± 8.442 10^−6^	75	20400	12.8	14.4
HsLaRP4A La-module	22.52 ± 0.60	0.0012 ± 2.5 10^−5^	21.66 ± 0.16	0.001151 ± 8.423 10^−6^	68	27800	17.4	20.5
HsLaRP4B La-module	21.33 ± 0.29	0.0018 ± 2.8 10^−5^	21.37 ± 0.03	0.01821 ± 1.937 10^−5^	67	28900	18.1	21.6
**Software employed**								
Primary reduction		Foxtrot						
Data processing		ATSAS 2.8 and Scatter						
Ab initio analysis		DAMMIF/DAMMIN						
Validation and averaging		DAMAVER						
Computation of model intensities		CRYSOL						
3D graphic representation		PyMOL

Overall, our SEC-SAXS data suggest that in solution all the apo La-modules examined are monomeric and populate non-globular conformations. Nevertheless, they exhibit varying degrees of intrinsic flexibility, with the following rank order: HsLaRP4A > HsLa > HsLaRP6 > HsLaRP4B. HsLaRP7 La-module cannot be confidently placed in this list because in the construct used (spanning residues 1–208), flexible residues at both N- and C-terminal ends of the La-module (residues 1–28 and 189–208, respectively) are likely to interfere with the analysis.

### RNA binding affects the hydrodynamics of the La-module of HsLa, HsLaRP6 and HsLaRP7

Next, we investigated the hydrodynamic properties of the La-modules when bound to RNA. We did not perform this analysis with HsLaRP4A and HsLaRP4B La-modules. Our recent report revealed that unexpectedly, in HsLaRP4A La-module plays only a minor role in the recognition of its target, polyA RNA. Instead, the main determinants of the interaction are located in the disordered N-terminal region, which surprisingly lacks discernible RNA-binding motifs [18]. The role of the HsLaRP4B La-module in RNA recognition is still under investigation (Conte, unpublished). For HsLa, HsLaRP6 and HsLaRP7, cognate RNAs that bind to the La-module with high affinity have been identified and extensively characterized, namely short 3ʹ oligoU sequences (U4) for HsLa and HsLaRP7 and the stem loop from the 5ʹUTR collagen mRNA (48ntSL) for HsLaRP6 [5–7]. SEC-SAXS was performed on these complexes (Fig. 1, red curves). The size exclusion profiles for the LaRP-RNA mixtures show two peak clusters: a single peak at lower retention volume, assigned to the LaRP-RNA complex and clusters at higher retention volume attributed to the excess RNA ligand used to prepare the samples. Notably, the aggregation observed for HsLaRP7 La-module in the apo state disappeared upon RNA binding. The higher UV absorption at 260 nm of the second peak cluster in the HsLa and HsLaRP7 profiles positively assigns them to free U4 in excess (data not shown). Indeed, incubation with a larger amount of oligonucleotide only increased the size of the second peak (not shown). For the HsLaRP6-48ntSL mixture, the CHROMIXS software [35] was used to examine the SAXS signal corresponding to the second elution peak, which regrettably elutes at the same retention time as the unbound HsLaRP6 La-module. This analysis revealed similar parameters to those obtained for the free 48ntSL RNA, thus positively ascribing the second peak to the surplus of RNA used in sample preparation. Interestingly, the SEC profile of the 48ntSL RNA alone did not generate a single peak profile, plausibly reflecting the existence of multiple conformations of this RNA molecule in solution (Supplementary Table S1 and Fig. S4).

Averaged SAXS curves, corrected for the solvent effect, were obtained for the La-module-RNA mixtures (Fig. 1F–H, red curves) selecting the frames corresponding to the protein-RNA complexes (Supplementary Fig. S2). Interestingly, for HsLa and HsLaRP7 the complexes show slightly increased retention times on the SEC-column compared to the free La-module, indicative of a configuration alteration and a compaction of these La-modules upon RNA binding (Fig. 1A,B). The adoption of a more compact conformation was substantiated by the hydrodynamic parameters (R_g_ and D_max_) (Table 1 and Fig. 2). Consistent with this, the normalized Kratky plots of the RNA-bound states (Fig. 1K–L, red curves) show a narrower distribution compared to the apo protein counterparts and the plateau region of the Porod-Debye plot is also reached earlier in the bound state (Supplementary Fig. S3A-B). This implies that HsLa and particularly HsLaRP7 La-modules achieve a more globular and rigid structure upon RNA binding (see discussion).

In the case of HsLARP6, the complex with 48ntSL RNA gives rise to a slightly broader Kratky profile (Fig. 1M, red curve), likely reflecting the contribution of this large RNA molecule to the overall shape of the protein-RNA complex. Similarly, in terms of flexibility, it is difficult to appreciate any change in the HsLaRP6 La-module upon RNA binding, which in the Porod-Debye plots translates in a more flexible profile for HsLaRP6-48ntSL complex, probably due to the intrinsic flexibility of the large RNA (Supplementary Fig. S3C).

### SAXS-derived low-resolution models for LaRP La-modules

Using the low-q region of the SAXS curves of the La-modules and their RNA complexes, theoretical distributions of the internal distances (P(r)) were obtained (Fig. 2A–E). Upon complex formation, the P(r) distribution is narrower for HsLa and HsLaRP7 showing a smaller D_max_ value compared to the respective apo La-modules, substantiating compaction of these La-modules upon RNA binding. For HsLaRP6, the large size of the bound RNA significantly influences the P(r) function, which differs considerably from that of both the free RNA and HsLaRP6 La-module (Fig. 2C).

With DAMMIF and DAMAVER, the P(r) distribution was used to calculate low-resolution *ab-initio* 3D envelopes representing the average of the ensemble of conformations explored by the molecules in solution (Fig. 2F–M) (see Methods). The *ab-initio* models of the La-modules in the absence of RNA (Fig. 2F–J) show non-globular shapes with two distinctive lobes of different sizes in all cases. Interestingly, albeit dissimilar, the envelopes of HsLa, HsLaRP7, HsLaRP6 and HsLaRP4B all recall a V-shaped arrangement of the two lobes, whereas for HsLaRP4A a more extended configuration is revealed (Supplementary Fig. S5 and S6, Fig. 2). Notably, and in agreement with the behaviour observed in the normalized Kratky plots and the P(r) distribution, the *ab-initio* models emphasize a compaction of HsLa and HsLaRP7 in the presence of oligoU4 RNA to a more globular shape, with a loss of the distinct lobe delineation (Fig. 2K,L). For HsLaRP6, the envelope in the presence of RNA changes dramatically (Fig. 2M), because of the significant contribution from the large 48ntSL RNA (Fig. 2N) to the overall shape of the protein-RNA complex.

A powerful strategy to evaluate the robustness of SAXS-derived envelopes is the comparison of the SAXS experimental data with theoretical SAXS curves back-calculated from available high-resolution structures, using the CRYSOL software included in the ATSAS package [29]. Such an analysis also provides information on the extent to which the behaviour of the molecules in solution is represented by the high-resolution models, as indicated by the χ^2^ value that for excellent fits would be around one. To test the methodology and gaining confidence in data interpretation, we performed CRYSOL analysis on the isolated LaM and RRM1 domains of HsLaRP4A and HsLaRP6 for which we have NMR structures [7,18] and were able to acquire high-quality SEC-SAXS datasets (Supplementary Fig. S7). These single domains all behave as globular molecules and produce good fits with CRYSOL (Fig. S5 F-I, Table 2 and Supplementary Table S1).

**Table 2.** CRYSOL and EOM analysis of the SAXS data for the La-modules using available high-resolution structural information

	CRYSOL		EOM	
SAXS data	Structural model	χ^2^	Rigid body model definition	χ^2^
HsLa La-module-U4	X-ray Structure PDB 2VOP	1.8	-	-
HsLa La-module	X-ray Structure PDB 2VOP (with the RNA removed)	7.1	PDB 2VOP; dummy residues: 99–107	1.03
HsLaRP7 La-module-U4	X-ray Structure PDB 4WKR	≫10	-	-
HsLaRP7 La-module	X-ray Structure PDB 4WKR (with the RNA removed)	≫10	PDB 4WKR; dummy residues: 1–27, 117–121 and 189–208	1.30
HsLaRP6 La-module-48ntSL	No structure available	-	-	-
HsLaRP6 La-module	No structure available	-	PDB 2MTF for LaM (70–178) and 2MTG for RRM1 (181–195); dummy residues: 178–179 and 296-300	2.55
HsLaRP6 LaM	NMR structure PDB 2MTF (removing residues 70–84)	1.2	-	-
HsLaRP6 RRM1	NMR structure PDB 2MTG	1.2	-	-
HsLaRP4A La-module	NMR structure PDB 6I9B	1.5	PDB 6I9B; dummy residues: 117–121 and 275-287	1.01
HsLaRP4A LaM	NMR structure PDB 6I9B	1.6	-	-
HsLaRP4A RRM1	NMR structure PDB 6I9B	2.2	-	-
HsLaRP4B	No structure available	-	-	-

CRYSOL analysis using the NMR representative structure of apo HsLaRP4A La-module [18] gave a χ^2^ value of 1.5 (Table 2 and Fig. S5C). Analysis on the dataset of HsLa bound to RNA, using the crystal structure of an HsLa-U4 complex, generated a relatively good fit (Fig. S5D and Table 2). On the contrary, a poor fit was obtained for HsLaRP7 in complex with U4 RNA (χ^2^ ≫10, Fig. S5E and Table 2). This is not surprising when considering the two stretches of residues (28 at the N-terminus and 19 at the C-terminus) that could not be seen in the electron density map [6], presumably because of their flexible nature. We currently lack high-resolution structures of HsLa, HsLaRP7, HsLaRP6 and HsLaRP4B La-modules in isolation and HsLaRP6 in complex with cognate RNA, hindering the CRYSOL analysis for the rest of the SAXS datasets. A speculative analysis was attempted for the La-modules of HsLa and HsLaRP7 in absence of RNA, by using models derived from the structures of their complexes but with the RNAs omitted. This generated large χ^2^ values, *i.e.* 7.08 for HsLa (PDB 2VOP) and ≫10 for HsLaRP7 (PDB 4WKR), thus demonstrating that the RNA-bound structures are not a good representation of the conformations of the molecules in solution in absence of RNA. This is highly consistent with the large differences observed for the SAXS-derived envelopes and the *ab-initio* models of the apo *versus* RNA-bound La-modules.

### Ensemble optimization method (EOM) describes the ensemble of La-module conformations in equilibrium

To investigate further the inherent molecular flexibility of the La-modules in solution in the absence of RNA revealed by our SAXS data, we used the Ensemble Optimization Method (EOM) [31]. With this approach we sought to generate molecular models representing the conformational sampling experienced in solution by the various La-modules that satisfy the SAXS experimental data. This method does not require high-resolution structures of the La-modules, but only the SAXS curves of the La-modules together with structural information of the individual domains (LaMs and RRM1s), that are available for all the LaRPs in this study except for HsLaRP4B, which was therefore not included in the analysis.

The overarching hypothesis of our EOM approach is that the La-module can be represented as made of two globular rigid domains (LaM and RRM1) linked by flexible dummy residues. Several observations to date corroborate this premise. First, the LaM and RRM1 appear to be structurally independent domains: they can be produced as isolated domains for HsLa, HsLaRP6 and HsLaRP4A for structural and functional characterization [4,7,18,23], and behave as monodisperse molecules in solution (see above). Furthermore, previous NMR analysis of the La-module of HsLa and HsLaRP4A suggests that in the absence of RNA the LaM and RRM are motionally independent and that the interdomain linker is a flexible portion of the protein [5,17,18]. To date, we do not have data on the behaviour in solution of the LaM and RRM1 domains of HsLaRP7 in isolation, although common features shared with HsLa, together with SAXS measurements shown in Fig. 1, endorse the view that such characteristics can also be applicable to HsLaRP7.

Residues of the interdomain linker that were left unrestrained (dummy residues) during the rigid body modelling were carefully selected from prior NMR structures and relaxation data for HsLa, HsLaRP6 and HsLaRP4 apo La-modules and/or LaM/RRM1 [4,5,7,17,18] (Table 2). For HsLARP7, the boundaries of the interdomain linker were deduced from the crystal structure in complex with the RNA [6], with the assumption that, analogously to HsLa, the α-helix present here would not possess a high degree of flexibility in the apo form, given its extensive contacts with the RRM1. Dummy residues were also attributed to regions of the La-modules which were either not observed in the crystal structure or known to experience intrinsic motion from NMR characterizations, specifically, residues at the N-and C-terminus of HsLaRP7 (1–28 and 189–208, respectively) [6] and C-terminal stretches for HsLaRP6 and HsLaRP4A (296–300 and 275–287, respectively) [7,18] (Table 2).

**Figure 3.**
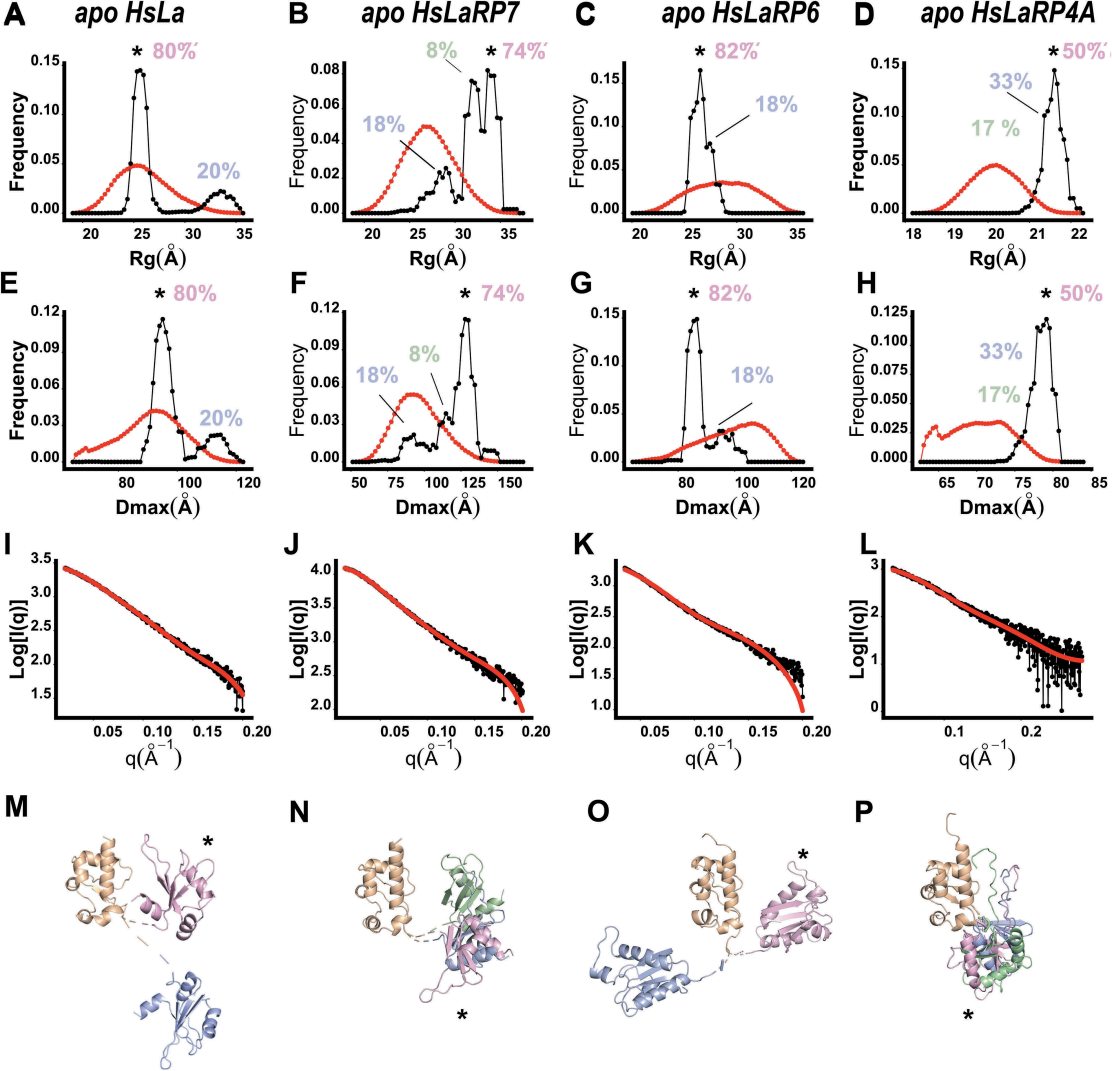
Ensemble Optimization Method (EOM) for the HsLa, HsLaRP7, HsLaRP6, and HsLaRP4A La-modules. (A-D) Distribution of the R_g_ (red) of the 10.000 conformations generated by allowing flexibility to the linker residues (see methods) and R_g_ for the ensemble of models that best fit the scattering data (*black*). The most populated state is marked with an asterisk (*) and the population percentages are shown. (E-H) Distribution of the D_max_ of all possible conformations (red) and those of the ensemble that best fit the scattering data (black). The most populated state is marked with an asterisk (*) and the population percentages are shown. (I-L) Fitting of the representative structures of the ensemble to the experimentally recorded scattering curves. (M-P) Representative structures of the ensemble of conformation in the context of the *ab-initio* models generated by DAMMIF, where the LaM domain (*orange*) is shown in a fixed orientation, to highlight the different positioning of the RRM1 domain (*in pink, blue and green*). The colour code used is: pink, blue, green from the most to least populated conformation. The most populated conformation of the RRM1 (*pink*) is marked with an asterisk (*). The linker regions, input as flexible residues in the EOM process, are represented as dashed lines

The results from the EOM analysis for HsLa, HsLaRP7, HsLaRP6 and HsLaRP4A La-modules in the apo form are reported in Fig. 3 and Table 2. In all cases, the ensembles of structures that best represent the experimental SAXS data are distributed in a relatively narrow window of R_g_ and D_max_ values. In other words, although the interdomain linkers are modelled as unrestrained, the tandem La-module configurations that best represent the behaviour of the molecules in solution appear to be restricted to a few distinct states in equilibrium. Moreover, interestingly, differences can be observed for the various La-modules investigated. For HsLa, the selected models are grouped in two structural clusters (Fig. 3A,E,I and M): the most populated of these (80% of the total population, R_g_ 27 Å, D_max_ 70 Å) shows similar hydrodynamic properties to the RNA-bound HsLa La-module (R_g_ 23 Å, D_max_ 69 Å), whereas the second conformation is more extended (20%, R_g_ 33 Å, D_max_ 80 Å, Fig. 3M). The EOM analysis for HsLaRP6 La-module indicates that this protein exists predominantly (82%) in a single subset of configurations (R_g_ 23 Å, D_max_ 72 Å) in the more compact range of possible conformations of the pool generated (Fig. 3C,G,K,O). On the other hand, the ensemble of representative models that best describes the SAXS data of HsLaRP7 and HsLaRP4A shows a wider distribution characterized by larger R_g_ and D_max_ values (R_g_ 31 Å, D_max_ 100 Å for HsLaRP7 and R_g_ 21.5 Å, D_max_ 80 Å for HsLaRP4A) (Fig. 3B,F,J and D,H,L respectively). This preference is particularly clear for HsLaRP4A, suggesting that the RRM1 explores discrete states in the conformational space whilst remaining in an overall extended configuration (Fig. 3P). For HsLaRP7, preferred LaM/RRM1 orientations in the absence of RNA are more extended compared to HsLa but less so than HsLaRP4A (Fig. 3N).

On the whole, the EOM analysis reveals a thus far overlooked ability of La-modules to populate distinct conformations in their apo form: on one side of the spectrum, HsLa and HsLaRP6, although intrinsically flexible, tend to populate more compact conformations in equilibrium, whilst HsLaRP4A, and possibly HsLaRP7, also flexible, prefer more extended LaM/RRM1 spatial arrangements.

### Discussion

Although La-modules share convergent features across the LaRP superfamily, they are astoundingly able to recognize distinct RNA targets [2]. With the exception of the wing2 region, the La-module sequence conservation is high in the LaM, while interdomain linkers and RRM1s greatly differ [2]. Notably, the interdomain linkers have been recognized as the most divergent portions of the La-module, with variable lengths and sequences, and although several lines of evidence corroborate their involvement in RNA-binding substrate recognition [2,5–8,17,18], their precise roles remain to be understood. Indeed, our knowledge of the RNA-binding mechanism by members of the LaRPs superfamily is still inadequate and/or fragmented by the limited availability of high-resolution structures in the absence and in the presence of their cognate RNAs. Structural studies have often been hampered by the intrinsic flexibility and/or poor solubility of the La-module and their complexes, making it challenging to yield samples amenable for X-ray crystallography or NMR characterizations. Moreover, static structures of protein-RNA complexes fall short of capturing the binding dynamics, obscuring the mechanistic connection between interface flexibility and ligand binding selection. In the present work we have therefore applied SEC-SAXS methodologies to reveal the molecular behaviour and conformational properties of a subset of human La-modules in solution.

The SAXS data shown here demonstrate that all the La-modules investigated behave as flexible tandem-domain proteins in solution in the apo state, albeit, importantly, the extent of their intrinsic motion varies considerably from highest in HsLaRP4A to lowest in HsLaRP4B. Furthermore, the flexibility of apo La-modules does not tie in with free exploration of the entire conformational space, as indicated by a few distinct and protein-specific populated states that exist in equilibrium.

**Figure 4.**
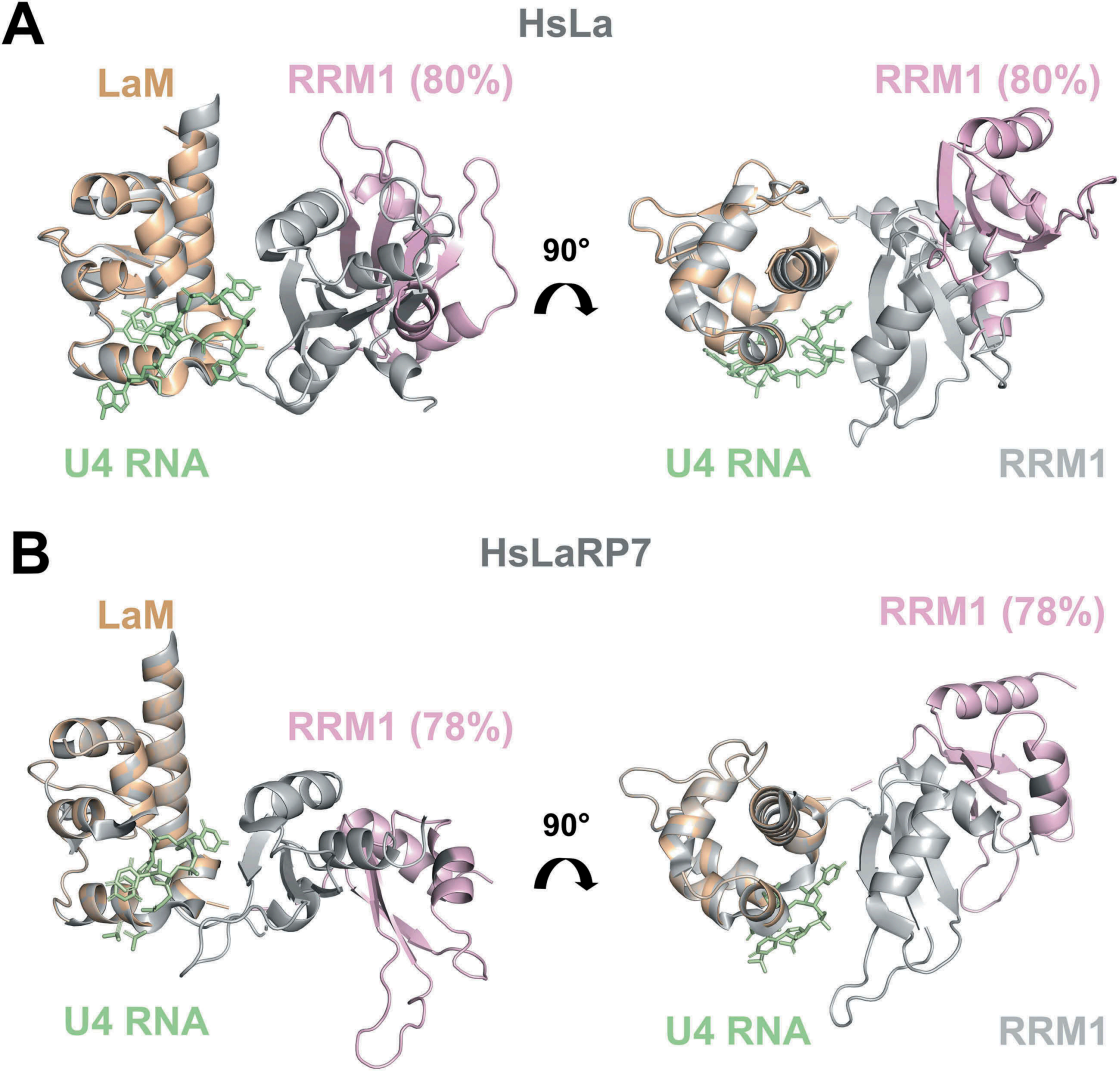
Comparison of crystal structures of RNA-bound forms with EOM-derived models of apo La-modules. (A) Superposition of the X-ray structure of HsLa-U4 (PDB 2VOP, protein in grey and RNA in green) with the most abundant apo HsLa EOM ensemble structure (LaM in orange and RRM1 in light pink, same colour-code used in Fig. 3). (B) Superposition of the X-ray structure of HsLaRP7-U4 (PDB 4WKR, protein in grey and RNA in green) with the preferred apo HsLaRP7 EOM ensemble structure (LaM in orange and RRM1 in light pink). The percentages indicate the fraction of the molecular population that exists in a particular state. The structures in (A) and (B) are aligned on the LaM. Although their RNA-bound forms are highly similar, the apo EOM models of HsLa and HsLaRP7 differ. For HsLa a more similar domain arrangement in the RNA-bound crystal structure and in the EOM model of the apo La-module could be observed (see also Supplementary Fig. S8)

Our investigations show that population equilibrium of HsLa, HsLaRP7 and HsLaRP6 responds to the presence of cognate RNA, and this is to our knowledge the first observation of such a behaviour for HsLaRP7 and HsLaRP6. The comparison of apo and RNA-bound states of HsLa and HsLaRP7 reported here unambiguously reveals a loss of flexibility coupled with compaction experienced by the La-module upon RNA binding. This thoroughly agrees with existing X-ray and NMR data of HsLa showing that the tethered LaM and RRM1 lock themselves into a compact V-shaped configuration in the complex with RNA [5,6,17]. In the absence of RNA, NMR relaxation experiments on HsLa demonstrated that the LaM and RRM1 are able to tumble independently without adopting a fixed relative orientation in solution [5]. Nonetheless, no information on a global shape and tandem orientation of the apo HsLa La-module could be derived by the NMR analysis alone [36]. Our SAXS investigations address this question: *ab-initio* calculations and EOM analysis show that, albeit distinct from the holo form, a two-lobed compact architecture is also adopted by HsLa La-module in the absence of RNA. The LaM and RRM1 in apo HsLa do not sample all the possible configurations, but only a restricted subset, with the most populated conformation being, intriguingly, quite compact (Fig. 4). In other words, while the LaM and RRM1 of apo HsLa are able to move with respect to one another in solution and are connected by a largely flexible tether [5], their relative spatial arrangement appears pre-set prior to the RNA encounter.

A global architectural organization similar to HsLa is adopted by the La-module of HsLaRP7 when bound to a 3ʹ-UUU ligand, but no information is available on its apo form from other sources [6]. Our SAXS data and analysis strongly argue for concomitant reduced flexibility and increased compaction of HsLaRP7 La-module upon RNA complex formation. Moreover, the population-weighted configurational ensemble of HsLaRP7 in the absence of RNA differs from HsLa (Figs. 3B,F and 4). This is interesting and somewhat surprising, given the many shared features between these two proteins.

SAXS is an excellent technique for elucidating the structural dynamics of flexible modular proteins when high-resolution information is available for the individual domains, and this was exploited here to investigate HsLaRP6 and HsLaRP4A La-modules. Regrettably, the large and flexible 48ntSL RNA ligand prevented a full characterization for HsLaRP6, given that it accounts for a large portion of the scattering envelope in the HsLaRP6-48ntSL complex; yet, results from CRYSOL and EOM indicate a V-shaped arrangement of the LaM and RRM1 in the context of the unbound protein and a distinctly compact character for the most populated conformation in solution, similar to what was observed for HsLa. Although the interaction of HsLaRP6 La-module with this ligand has been extensively studied [7,37], it has remained challenging to establish the exact role of HsLaRP6 RRM1 in this recognition mechanism [7]. Comparison of the data from the RNA alone and the *ab-initio* model of the HsLaRP6-48ntSL complex suggests a large interaction surface between the RNA and the protein, highly likely to include the RRM1 portion of the La-module; nonetheless, since SAXS cannot provide atomic resolution information, a precise role of the RRM1 cannot be conclusively assigned from our study, other than endorsing its necessary involvement in RNA binding as previously reported [7].

The tandem domain flexibility of HsLaRP4A La-module observed in the SAXS analysis is entirely consistent with previous NMR investigations [18,23]. A narrow window of populated conformations in solution is however revealed here, indicating that, alike the other La-modules, HsLaRP4A La-module does not sample the entire conformational space. Yet, intriguingly, the populated configurations for HsLaRP4A delineate a highly dissimilar architecture from all the other La-modules analysed in this study. The inability of HsLaRP4A La-module to bind to oligoA [18] may, therefore, pose the question as to whether such an extended conformation correlates with RNA-binding proficiency. As for HsLaRP6, the interdomain linker of HsLaRP4A is very short; however, the lack of wing2 in HsLaRP4A LaM presumably accounts for the different conformational space explored by these two LARPs (compact *versus* extended) [18].

Based on these results, it is tempting to speculate that a wing2/linker-driven restricted sampling of the conformational space is a conserved property of the La-modules in the absence of RNA, and that the differences in the wing2 and/or linker regions (both in length and sequence) infer the distinct weighted populations adopted by each protein in the unbound state. This, in turn, plausibly modulates their RNA-binding properties. Hence, although the LaM wing2 does not directly contact the nucleic acid ligand in contrast to other winged-helix domains [38], it may still tune RNA recognition *via* affecting the structure and conformational dynamics of La-modules. Our findings may, therefore, pave the way for the design of new experiments to unravel the role of wing2 and interdomain linker in enabling cooperativity and combinatorial binding by the LaM and RRM1.

Our SAXS data unambiguously reveal that a closed conformation of apo HsLa pre-dominates even in the absence of the RNA ligand (Fig. 4), and this is notable because association with RNA was thought to anchor the LaM and the RRM1 in the closed, RNA-binding competent V-shaped configuration, with the apo form adopting different unbound conformations in equilibrium and using its structural flexibility to select its RNA partner [2,5,16]. Data presented here however suggests that compact near-competent-like conformations of unbound HsLa La-module may facilitate molecular recognition of the target RNA, in line with a conformational selection-type mechanism [19,39,40]. Although somewhat burdened by additional flexible residues, SAXS analyses for HsLaRP7 suggest that the most populated unbound conformation of its La-module is less similar to the RNA-bound state compared with what was observed for HsLa, tentatively suggesting a higher contribution from an induced fit-type mechanism of recognition instead [19,39] (Fig. 4 and Supplementary Figure S8). This could, in turn, be favourable for a step-wise assembly of the 7SKsnRNP involving for instance the HsLaRP7 C-terminal region downstream the RRM2 which has been implicated in MePCE binding [41]. Such a hypothesis would require additional experimental evidence.

In conclusion, our SAXS data contributes to the dynamic pathway description for a few La-modules from the apo to the bound state. Whilst awaiting for further experimental data, it might be hypothesized that the wing2 and interdomain linker would not only enable the correct LaM/RRM1 orientation to generate the RNA-binding surface, but also dictate the mechanism of RNA recognition by the La-modules, i.e. conformational selection, induced fit or a differently weighted combination of the two, which may play a crucial role for RNA target discrimination by these proteins. Furthermore, it could be argued that the preservation of a V-shape conformation of apo La-modules may be a conserved mechanism to enhance binding to their cognate RNA, since HsLaRP4A La-module, characterized by a fully extended shape, does not contribute much to the binding of its RNA target [18].

Taken together, our results propose a link between RNA recognition modes by LaRP La-modules and the complexity of their conformational-energy landscape. Thus, single structural images alone would be inadequate to describe such molecular recognition processes. Moreover, the determination of static structures from ensemble-averaged experimental data can miss vital conformational and dynamic information that may be critical for biological activity. This study, therefore, contributes to a deeper knowledge and understanding of the molecular aspects of LaRPs and suggests that the structural dynamics in the La-modules may be an important player in the RNA-binding versatility of this protein superfamily.

## Supplementary Material

Supplemental Material
