# Supplementary material for: Structural dynamics in the La-module of La-related proteins
Source: RNA Biol. 2020 Mar 18;18(2):194–206. doi: 10.1080/15476286.2020.1733799 (PMC7928032; doi:10.1080/15476286.2020.1733799)
Supplement: Supplemental Material [file KRNB_A_1733799_SM1838.docx]

**Structural dynamics in the La-module of La-related proteins**

Javier Lizarrondo^1^, Anne-Catherine Dock-Bregeon^2^, Luigi Martino^3^* and Maria R Conte^1^*

^1^Randall Centre for Cell and Molecular Biophysics, King’s College London, Guy’s Campus, London SE1 1UL, UK

^2^Laboratoire de Biologie Intégrative des Modèles Marins, Station Biologique de Roscoff, CNRS-Sorbonne Université, Roscoff, France

^3^The Francis Crick Institute, Molecular Structure of Cell Signalling Laboratory, 1 Midland Road, London NW1 1AT, UK.

*to which the correspondence should be addressed: [sasi.conte@kcl.ac.uk](mailto:sasi.conte@kcl.ac.uk); [l.martino@wellcome.ac.uk](mailto:l.martino@wellcome.ac.uk)

Current address:

Javier Lizarrondo: European Molecular Biology Laboratory (EMBL) Hamburg, Notkestrasse 85, D-22607 Hamburg, Germany

Luigi Martino: Wellcome Trust, 215 Euston Road, London NW1 2BE, UK.

**Supplementary material comprising Figures S1-S8 and Table S1.**


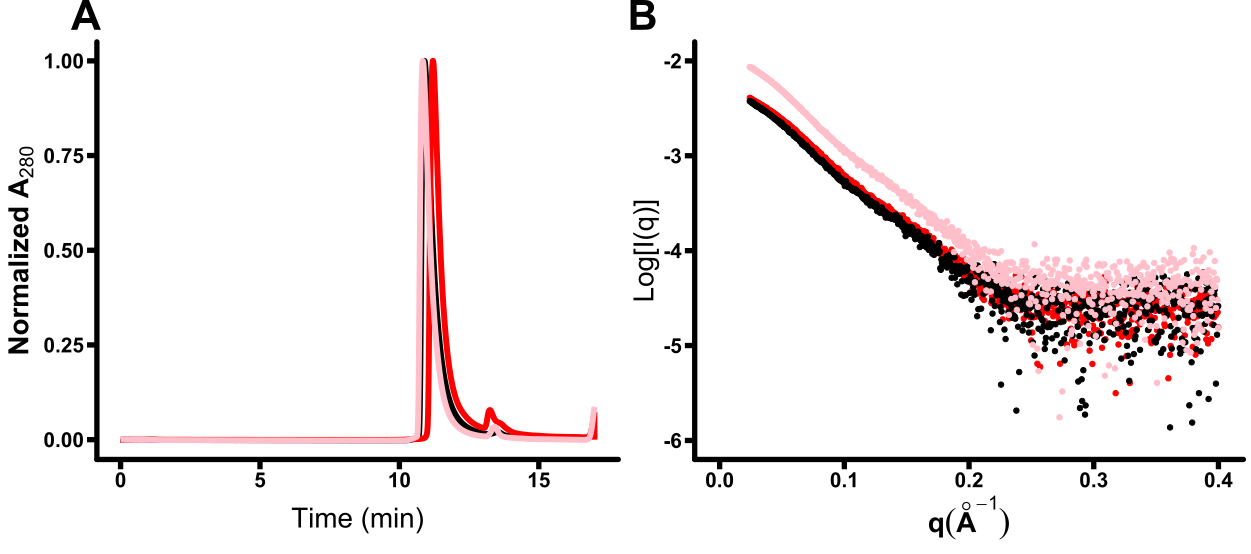


**Supplementary Figure S1*.***Size-exclusion chromatography (SEC) analysis of HsLaRP6 La-module constructs. **(A)** SEC elution traces for HsLaRP6 70-300 (black), 74-300 (pink) and 85-300 (red). **(B)** From the elution, SAXS curves were recorded and corrected for the scattering of the buffer.


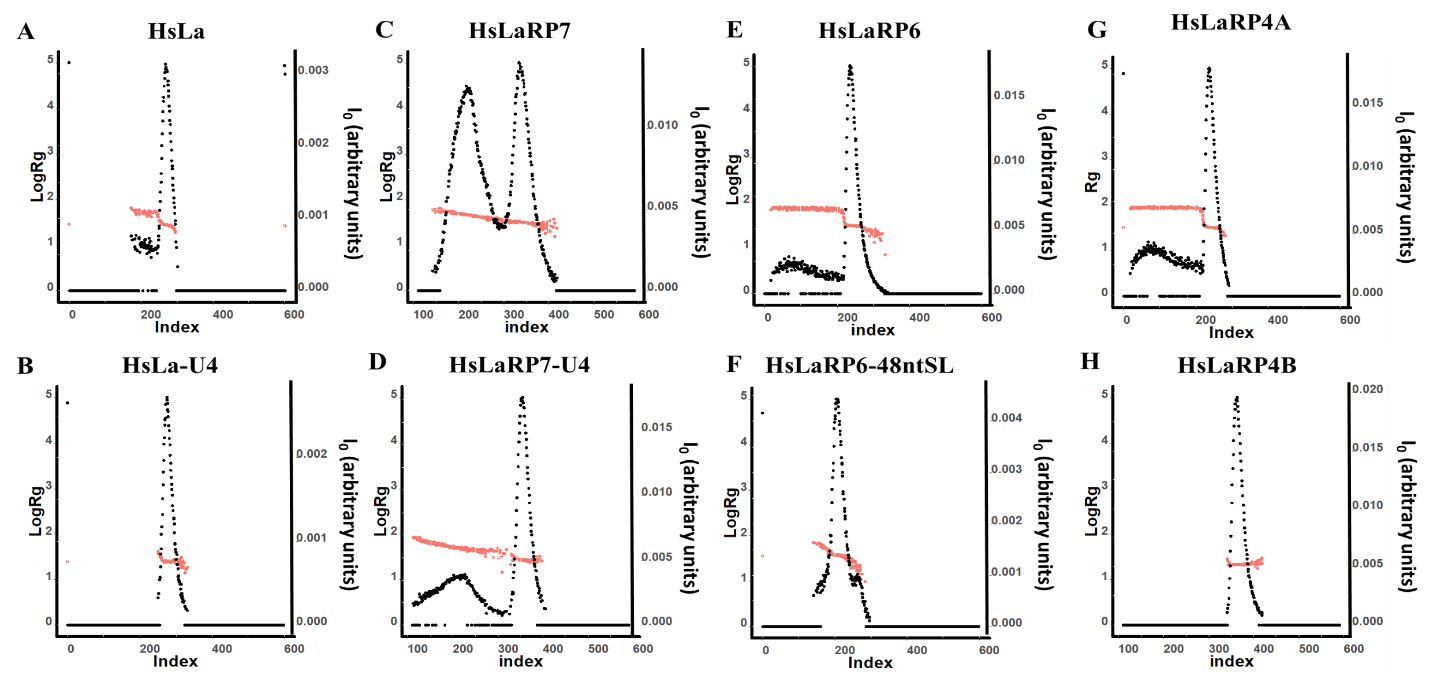


**Supplementary Figure S2.**Representation of the Log**(**R_g_) and the I_0_ across the SEC-SAXS frames collected for (**A**) HsLa La-module, (**B**) HsLa La-module-U4, (**C**) HsLaRP7 La-module, (**D**) HsLaRP7 La-module-U4, (**E**) HsLaRP6 La-module, (**F**) HsLaRP6 La-module-48ntSL, (**G**) HsLaRP4A La-module and (**H**) HsLaRP4B La-module. The most intense frames were averaged and buffer subtracted to obtain the SAXS curves for each La-module. The R_g_ is constant throughout the elution peaks indicating monodisperse samples from which the SAXS curves can be obtained.


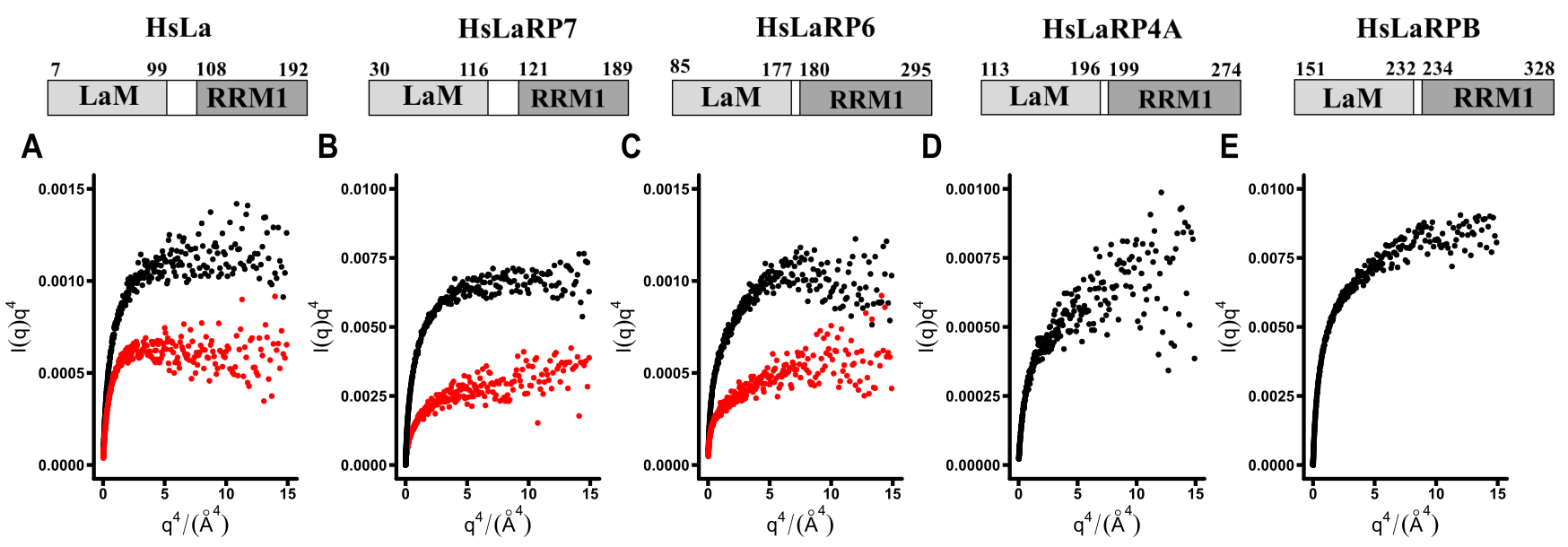


**Supplementary Figure S3**. Porod-Debye representation of the SAXS curves of the La-modules of HsLa, HsLaRP7, HsLaRP6, HsLaRP4A and HsLaRP4B. (**A-E**) Traces for the apo species are shown in black and those in complex with RNA (U4 for HsLa and HsLaRP7 and 48ntSL for HsLaRP6) are shown in red in **A**, **B** and **C** respectively.


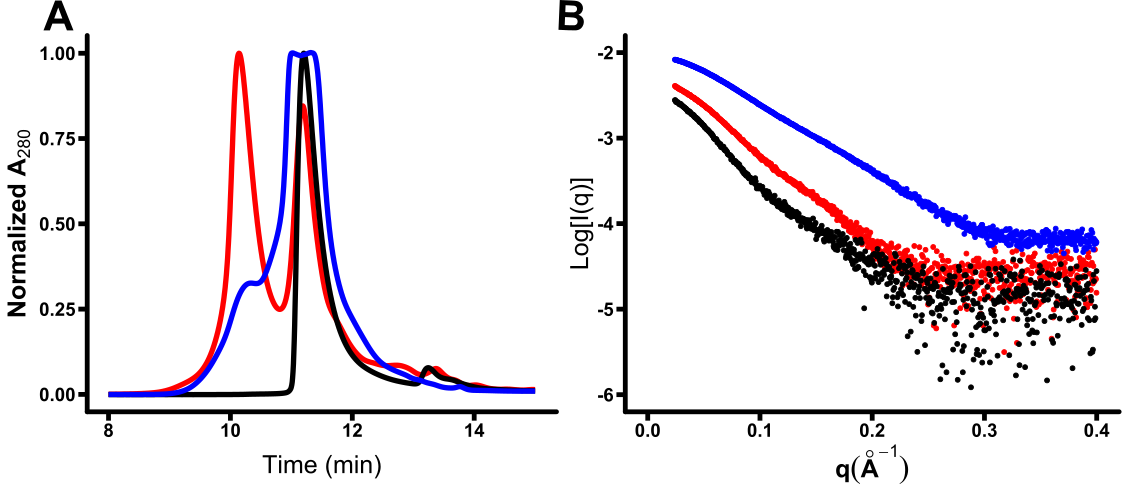


**Supplementary Figure S4.** Size-exclusion analysis of HsLaRP6 La-module, 48ntSL RNA and their complex. (**A**) Size exclusion traces obtained for HsLARP6 85-300 (black), 48ntSL RNA (blue) and the HsLARP6-48ntSL complex (red). (**B**) From the elution, SAXS curves were recorded and corrected for the scattering of the buffer.


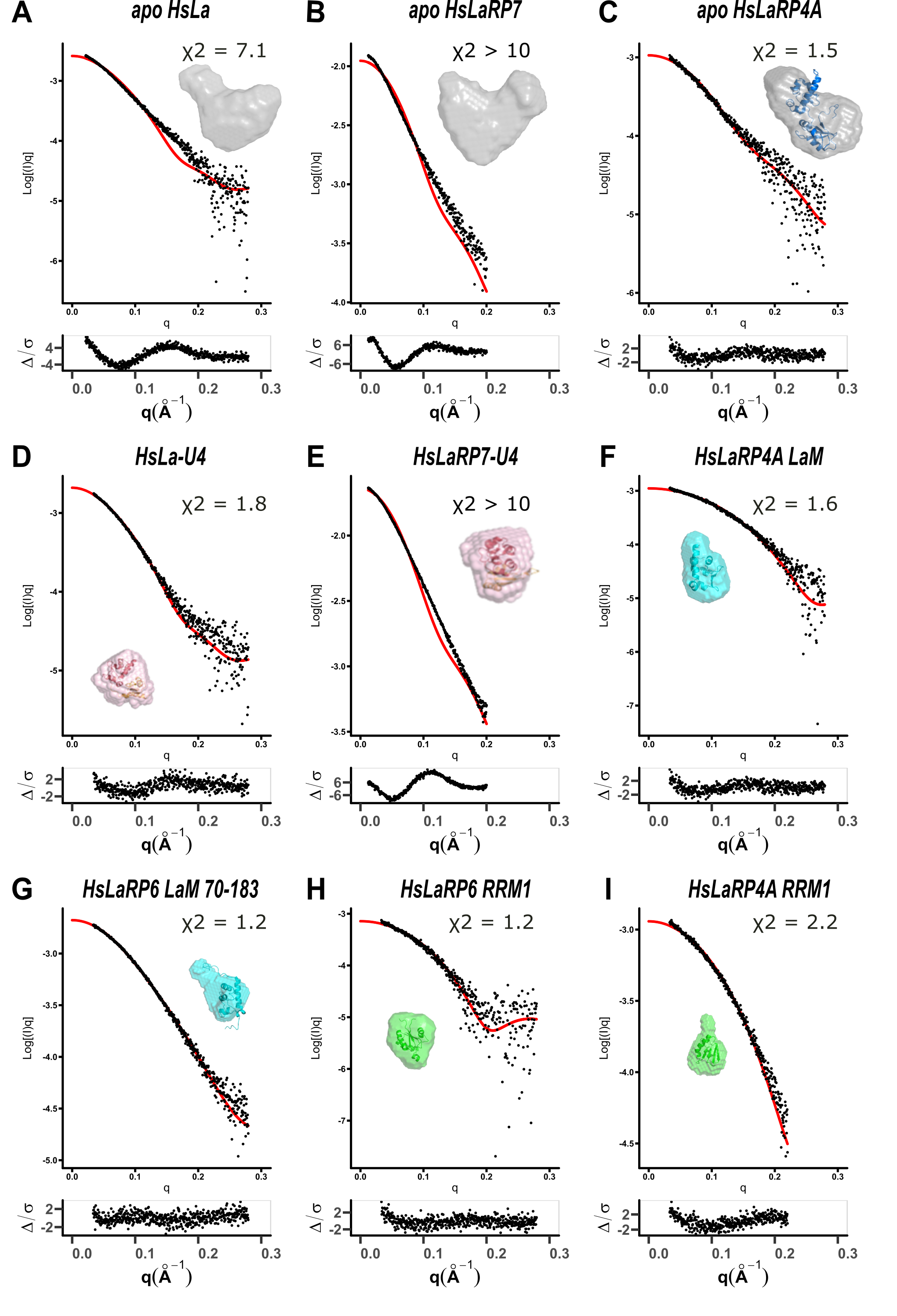


**Supplementary Figure S5.** CRYSOL fitting of the experimental SAXS curves to the available high-resolution structures for: (**A**) apo HsLa La-module (PDB 2VON with the RNA removed); (**B**) apo HsLaRP7 La-module (PDB ID 4WKR with the RNA removed); (**C**) apo HsLaRP4A La-module (PDB 6I9B); (**D**) HsLa La-module-U4 complex (PDB 2VON); **(E**) HsLaRP7 La-module-U4 complex (PDB ID 4WKR); (**F**) HsLaRP4A LaM (PDB 6I9B, residues 111-196);  (**G**) HsLaRP6 LaM (70-183) (PDB 2MTF); (**H**) HsLaRP6 RRM1 (PDB 2MTG); (**I**) HsLaRP4A RRM1 (PDB 6I9B, residues 199-297). The residual differences between the experimental and the calculated values of Log[I(q)] are reported in the lower panels for each fitting as the difference divided by the error (Δ/σ=(I(exp)-I(mod)/σ)).


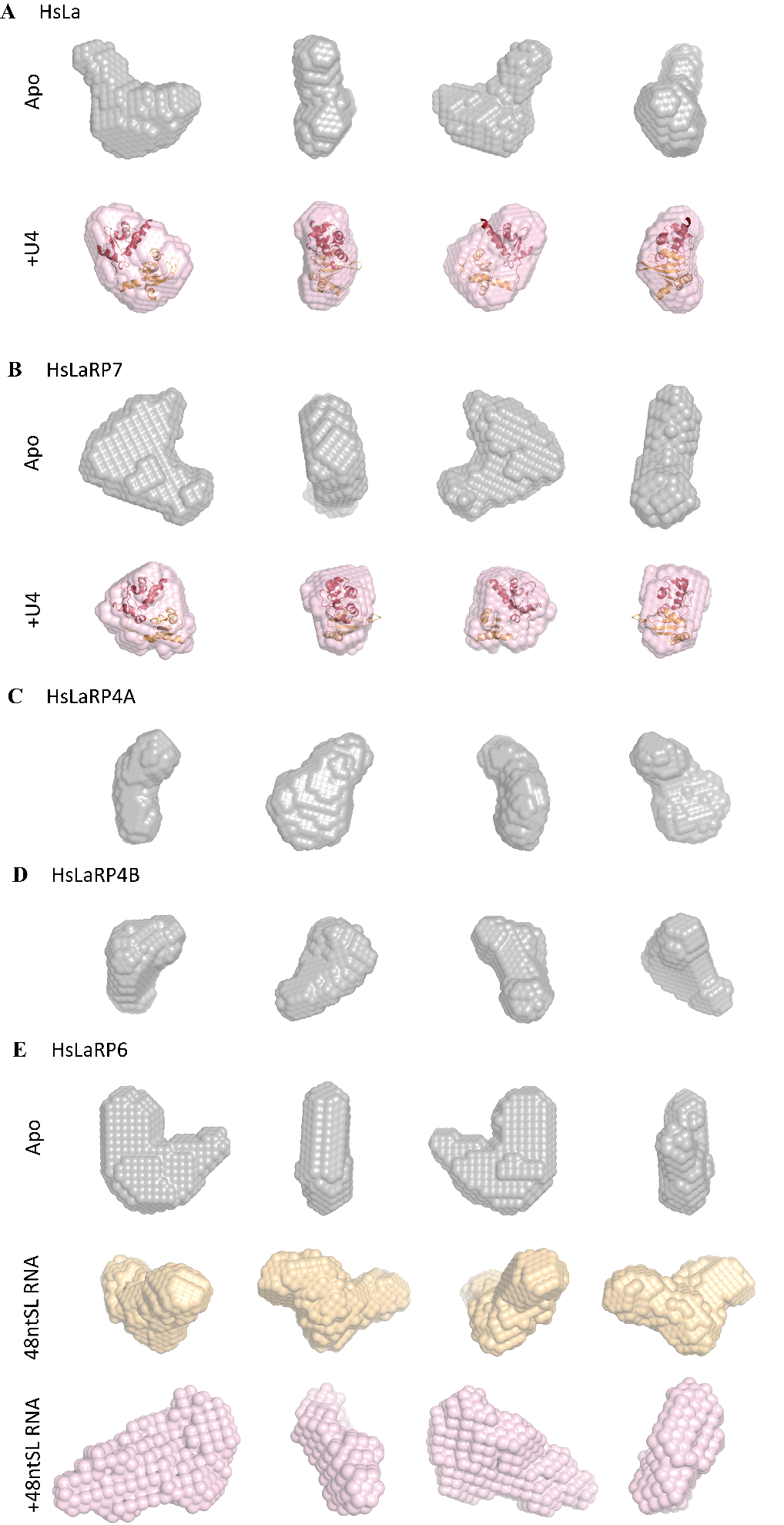


**Supplementary Figure S6.**  Different views of the *ab-initio* envelopes generated using DAMMIF for the La-modules of (**A**) HsLa, (**B**) HsLaRP7, (**C**) HsLaRP4A, (**D**) HsLaRP4B and (**E**) HsLaRP6. The envelopes were first fitted by SUPCOMB on the respective atomic structures of La-modules, where available (*i.e.* for HsLa, HsLaRP7 and HsLaRP4). For HsLaRP6 and HsLaRP4B, the envelopes were fitted on the HsLa model. Similar orientations to **A** and **B** were chosen for all envelopes (with the LaM domain on the left) and rotated by +90° along the Y-axis for each orientation shown. Arbitrary orientations were chosen for the 48ntSL RNA and HsLaRP6 complex. Fitted structures are only shown within the envelopes for the U4-bound HsLa and HsLaRP7 (bottom lines of (**A**) and (**B**)), where the relative fixed orientations of the LaM and RRM1 are known from crystal structures.


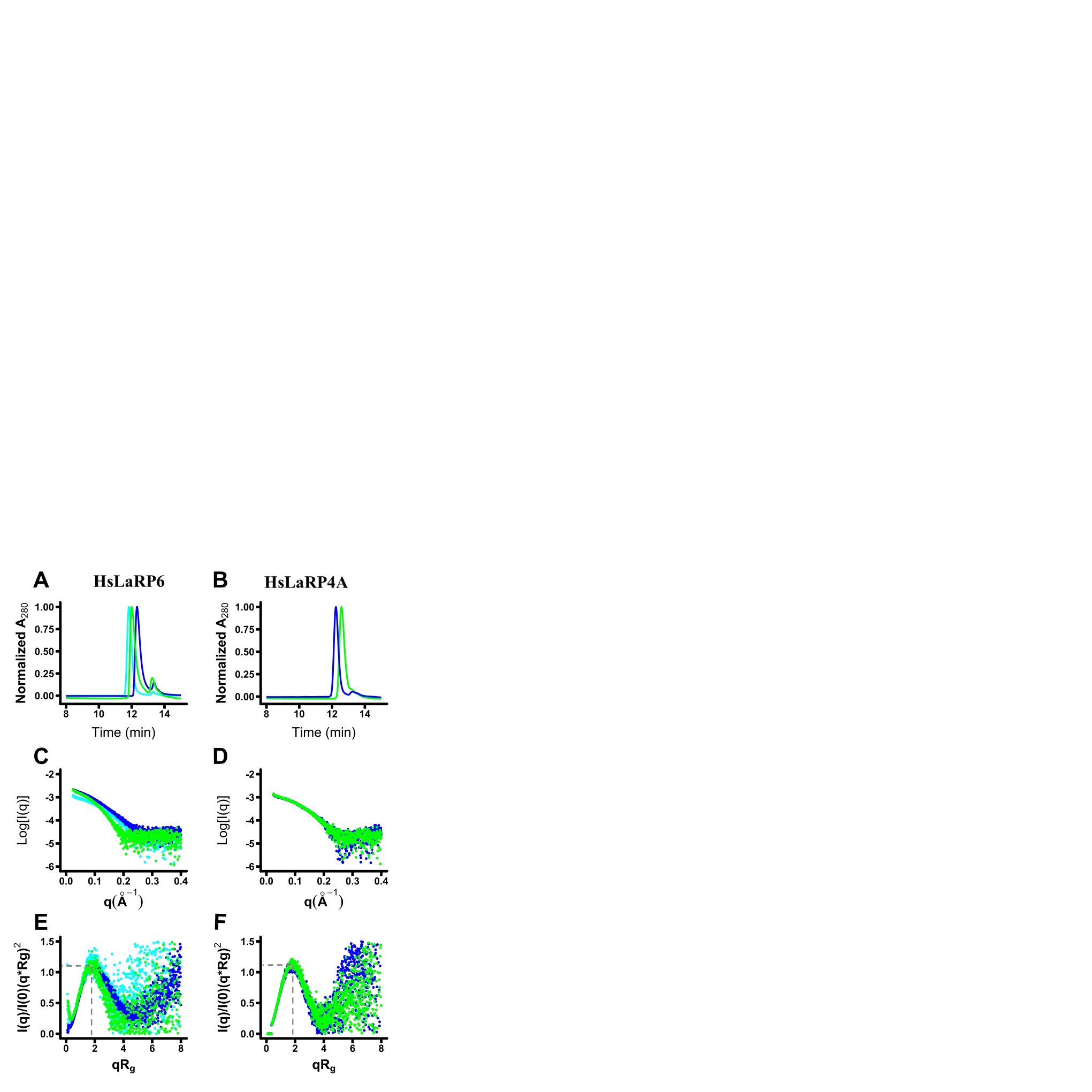


**Supplementary Figure S7**. SAXS analysis of the isolated LaM and RRM1 of HsLaRP6 and HsLaRP4A. (**A**) Size exclusion traces for HsLaRP6 LaM 70-183 (blue), HsLARP6 LaM 85-183 (light blue), and HsLaRP6 RRM1 (green); (**B**) size exclusion traces for HsLaRP4A LaM (blue) and HsLaRP4A RRM1 (green). All the domains migrated in the SEC column as monodisperse species. (**C,D**) SAXS curves recorded and obtained after buffer normalization from A and B respectively, using the same color scheme as A and B. (**E-F**) Normalized Kratky representation for C and D respectively, revealing that these domains behave as globular proteins. The typical values expected for globular proteins [I(q)/I(0)]⋅(q⋅R_g_)^2^=1.104, q⋅R_g_=1.73] are indicated by grey dashed cross lines.


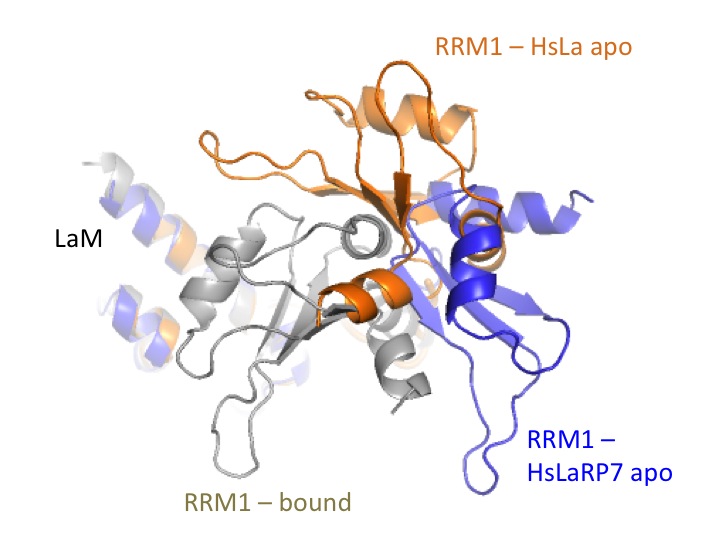


**Supplementary Figure S8**. Difference in the position of the RRM1 domain between HsLa and HsLaRP7 in the apo state. The most populated conformations obtained from the EOM analysis for HsLa apo (orange) and HsLaRP7 apo (blue) were superposed onto the RNA-bound conformation of HsLa (grey). The RNA-bound conformation of HsLaRP7 is not shown, being very similar to HsLa (see Fig. 4). The models were first superposed on the LaMs as in Fig. 4. The view is from the RRM1 side, with the LaM at the back. The spatial orientation of apo HsLa RRM1 is more similar to the RNA-bound state than HsLaRP7, implying that a larger conformational change would occur in HsLaRP7 upon RNA interaction.

**Supplementary Table S1: SAXS-derived parameters and experimental details**

| **Instrument** | | SEC-SAXS at SWING beamline SOLEIL | | | | | | | | |
| --- | --- | --- | --- | --- | --- | --- | --- | --- | --- | --- |
| **q range (Å^-1^)** | | 0.0022-0.62 | | | | | | | | |
| **Temperature (°C)** | | 25°C | | | | | | | | |
|  | | | | | | | | | | |
| **Sample** | **R_g_(Å) Reciprocal Space** | **I_(0)_**  **(cm^-1^/absorbance) Reciprocal Space** | | **R_g_(Å) Real Space** | **I_(0)_**  **(cm^-1^/absorbance) Real Space** | **D_max_**  **(Å)** | **GNOM Total Quality Estimate/ Type of solution** | **Porod Volume estimate**  **(Å^3^)** | **MW from Porod Volume/**  **1.6 (kDa)** | **MW from sequence (kDa)** |
| HsLa La-module | 26.90  ± 0.40 | 00029  ± 1.1 10^-5^ | | 26.97  ± 0.09 | 0.002876  ± 8.285 10^-6^ | 84 | 0.7928/  GOOD | 46400 | 29.0 | 22.3 |
| HsLa La-module-U4 RNA complex | 23.82  ± 0.25 | 0.0022  ± 1.5 10^-5^ | | 22.76  ± 0.08 | 0.002103  ± 6.678 10 ^-6^ | 69 | 0.7594/  GOOD | 40300 | 25.0 | 22.3 |
| HsLaRP7 La-module | 32.08  ± 1.02 | 0.0129  ± 2.6 10^-5^ | | 32.20  ± 0.10 | 0.01289  ± 2.96510^-5^ | 101 | 0.8419/  GOOD | 58200 | 36.4 | 24.0 |
| HsLaRP7 La module-U4 complex | 26.79  ± 0.30 | 0.0237  ± 3.5 10^-5^ | | 27.40  ± 0.05 | 0.02369  ± 3.196 10^-5^ | 92 | 0.7984/ GOOD | 48000 | 30.0 | 24.0 |
| HsLaRP7 La-module-U5 complex | 26.13  ± 0.23 | 0.011  ± 1.7 10^-5^ | | 26.64  ± 0.05 | 0.01068  ± 1.474 10^-5^ | 88 | 0.7965/  GOOD | 40200 | 25.1 | 24.0 |
| HsLaRP6 70-300 La-module | 30.57  ± 0.28 | 0.010  ± 7.3 10^-5^ | | 29.83  ± 0.06 | 0.01015  ± 2.428 10^-5^ | 89 | 0.8028/ GOOD | 62500 | 39.0 | 26.5 |
| HsLaRP6 70-300 La-module (1:2 dilution) | 29.77  ± 0.43 | 0.0028  ± 3.5 10^-5^ | | 27.92  ± 0.96 | 0.002621  ± 1.238 10^-5^ | 77 | 0.7498/  REASONABLE | 54200 | 33.9 | 26.5 |
| HsLaRP6 74-300 La-module | 28.67  ± 0.18 | 0.0042  ± 2.7 10^-5^ | | 29.27  ± 0.10 | 0.004246  ± 1.640 10^-6^ | 89 | 0.7606/  GOOD | 58100 | 36.3 | 26.4 |
| HsLaRP6 74-300 La-module (1:2 dilution) | 28.48  ± 0.29 | 0.0018  ± 1.8 10^-5^ | | 28.28  ± 0.10 | 0.001735  ± 9.220 10^-5^ | 84 | 0.9032/  EXCELLENT | 55400 | 34.6 | 26.4 |
| HsLaRP6 85-300 La-module | 27.18  ± 0.18 | 0.0045  ± 3.0 10^-5^ | | 27.20  ± 0.07 | 0.004412  ± 1.539 10^-5^ | 81 | 0.8261/  GOOD | 51900 | 32.4 | 23.9 |
| HsLaRP6 85-300 La-module-48ntSL RNA complex | 31.48  ± 0.13 | 0.0032  ± 1.8 10^-6^ | | 31.60  ± 0.07 | 0.003138  ± 1.220 10^-5^ | 90 | 0.9351/  EXCELLENT | 94000 | 58.8 | 39.8 |
| 48ntSL RNA | 22.29  ± 1.77 | 0.0091  ± 9.9 10^-6^ | | 22.47  ± 0.03 | 0.009076  ± 8.442 10^-6^ | 75 | 0.6993/  REASONABLE | 20400 | 12.8 | 14.4 |
| HsLaRP4A La-module | 22.52  ± 0.60 | 0.0012  ± 2.5 10^-5^ | | 21.66  ± 0.16 | 0.001151  ± 8.423 10^-6^ | 68 | 0.8296/ GOOD | 27800 | 17.4 | 20.5 |
| HsLaRP4B La-module | 21.33  ± 0.29 | 0.0018  ± 2.8 10^-5^ | | 21.37  ± 0.03 | 0.01821  ± 1.937 10^-5^ | 67 | 0.8896/ GOOD | 28900 | 18.1 | 21.6 |
| HsLaRP4B RRM1 | 15.37  ± 0.23 | 0.0012  ± 5.5 10^-6^ | | 14.95  ± 0.06 | 0.001190  ± 3.944 10^-6^ | 46 | 0.8694/  GOOD | 13600 | 8.5 | 10.8 |
| HsLaRP6 RRM1 | 21.71  ± 0.47 | 0.0021  ± 2.3 10^-5^ | | 19.09  ± 0.05 | 0.001929  ± 5.490 10^-6^ | 57 | 0.9404/  EXCELLENT | 31200 | 19.5 | 13.1 |
| HsLaRP6 RRM1 Diluted | 20.83  ± 0.87 | 0.00082  ± 1.8 10^-5^ | | 18.86  ± 0.09 | 0.0007635  ± 5.192 10^-6^ | 54 | 0.8505/  GOOD | 31500 | 19.7 | 13.1 |
| HsLaRP4A LaM | 20.93  ± 1.18 | 0.0013  ± 3.4 10^-5^ | | 14.45  ± 0.05 | 0.001146  ± 3.282 10^-5^ | 42 | 0.7342/  REASONABLE | 11600 | 7.3 | 10.1 |
| HsLaRP6 85-183 LaM | 17.47  ± 1.18 | 0.0011  ± 2.3 10^-5^ | | 15.82  ± 0.13 | 0.001057  ± 4.805 10^-6^ | 53 | 0.7508/  GOOD | 12500 | 7.8 | 11.1 |
| HsLaRP6 85-183 LaM (1:2 dilution) | 16.80  ± 0.45 | 0.0005  ± 7.1 10^-5^ | | 15.19  ± 0.10 | 0.000534  ± 7.440 10^-6^ | 46 | 0.7508/  GOOD | 12900 | 8.1 | 11.1 |
| HsLaRP6 70-183 LaM | 19.68  ± 0.77 | 0.0016  ± 2.5 10^-5^ | | 17.50  ± 0.06 | 0.001514  ± 4.259 10^-6^ | 54 | 0.7730/  GOOD | 15800 | 9.9 | 12.8 |
| HsLaRP6 70-183 LaM  (1:2 dilution) | 19.45  ± 0.57 | 0.0022  ± 3.0 10^-5^ | | 17.40  ± 0.05 | 0.002065  ± 5.242 10^-6^ | 53 | 0.8409/  GOOD | 16100 | 10.1 | 12.8 |
|  | | | | | | | | | | |
| **Software employed** | | | | | | | | | | |
| primary reduction | | | Foxtrot | | | | | | | |
| Data processing | | | ATSAS 2.8 and Scatter | | | | | | | |
| Ab initio analysis | | | DAMMIF/DAMMIN | | | | | | | |
| Validation and averaging | | | DAMAVER | | | | | | | |
| Computation of model intensities | | | CRYSOL | | | | | | | |
| 3D graphic representation | | | PyMOL | | | | | | | |
